# Editorial Comment to Synchronous bilateral renal cell carcinomas with differing histologies

**DOI:** 10.1002/iju5.12200

**Published:** 2020-07-27

**Authors:** Takeshi Kishida

**Affiliations:** ^1^ Department of Urology Kanagawa Cancer Center Yokohama Japan

Shigehisa *et al.* successfully treated synchronous bilateral renal tumors with different histology.^1^ The one was pT1b clear cell carcinoma, and the other was pT1a mucinous tubular and spindle cell carcinoma, both were treated by robot‐assisted partial nephrectomy (RAPN) sequentially in 3 months interval.

The significance of their case report is not only the rareness of the disease but also the treatment strategy for this specific situation. The development of RAPN enabled wider indication of nephron sparing surgery. For the bilateral kidney tumors, RAPN should be considered first to maintain the kidney function and minimum invasiveness. In performing RAPN for the both kidneys suspecting different histology, we must discuss about: What is the suspected histology based on the radiological examination? Is preoperative biopsy necessary? Do we operate sequentially or simultaneously? What is the appropriate interval? Which side should be operated first? Are there any options such as ablation therapy or active surveillance? There may be no concrete answer for these questions, however, we have to present many alternatives to the patients and inform them of the merits and demerits of each strategies. It is very difficult and time consuming effort but absolutely necessary. To treat the renal cell tumors we must have ability to perform many choices of procedures but it is impossible to experience every rare situation like this case. This case report will be very useful in the decision making in the similar circumstances.

## Conflict of interest

The author declares no conflict of interest.
